# Correlation of Zinc and Copper Levels In Mothers and Cord Blood of Neonates With Prematurity and Intrauterine Growth Pattern

**DOI:** 10.7759/cureus.63674

**Published:** 2024-07-02

**Authors:** Srinija Garlapati, Nagaraja Venigalla, Shailaja Mane, Amulya Dharmagadda, Kasireddy Sravanthi, Aryan Gupta

**Affiliations:** 1 Pediatrics, Dr. D. Y. Patil Medical College, Hospital & Research Centre, Pune, IND; 2 Pediatrics, Ankura Hospital for Women and Children, Vijayawada, IND; 3 Pediatric Neurology, Dr. D. Y. Patil Medical College, Hospital & Research Centre, Pune, IND

**Keywords:** trace elements, preterm delivery, intrauterine growth restriction (iugr), maternal copper, maternal zinc

## Abstract

Background

Trace elements like zinc and copper are indispensable for human growth and development, exerting significant influence on a multitude of physiological processes. Acting as pivotal components for transcription factors and catalytic cofactors for enzymes, these elements play essential roles in cellular differentiation and maturation

Objective

The objective of this study was to study serum zinc and copper levels in mothers and neonates in relation to prematurity and intrauterine growth retardation (IUGR).

Methods

This was a cross-sectional study that included 100 mothers who met the inclusion criteria. Maternal history was recorded, and gestational age was estimated using the New Ballard scoring system. Maternal and cord blood samples were taken for zinc and copper analysis.

Results

The comparison of maternal copper and zinc levels between term and preterm neonates revealed a statistically significant difference with both trace elements found in less concentration in preterm when compared to the term patients (p= 0.03 for Zinc; 0.0001 for copper). We also report a statistically significant difference in maternal and cord blood copper and zinc levels in cases with IUGR compared to normal neonates.

Conclusion

The findings show that maternal zinc and copper levels are critical for the intrauterine growth of the fetus and for appropriate gestational age

## Introduction

Infant mortality and morbidity risks rise as gestational age at birth decreases, particularly for very low-birth-weight (LBW) infants [1,2]. Preterm neonates, born before the 37th week of gestation, face elevated risks of poor outcomes due to incomplete micronutrient reserves, often resulting in low birth weight or being small for gestational age [3]. Zinc plays a crucial role in cell growth and development, being essential for RNA and DNA synthesis [4]. Severe zinc deficiency can lead to CNS impairments, including behavioral issues, decreased intelligence, and memory and learning difficulties [5].

In contrast, copper levels increase during pregnancy as gestational age progresses, driven by rising estrogen levels and ceruloplasmin production [6]. This ensures adequate serum copper levels for both the mother and the baby. Copper deficiency can negatively impact CNS and heart function during development, potentially leading to complications such as hearing loss and reduced motor function, which may persist despite replacement therapy [7].

Given the importance of zinc and copper in cell growth, maintaining sufficient levels of these elements is crucial for preterm and LBW neonates [1]. Maternal zinc restriction during fetal development in rodent models has been linked to intrauterine growth retardation [8]. However, studies have produced conflicting results regarding the impact of maternal zinc supplementation on outcomes such as fetal loss, fetal growth, and birth weight. Notably, the reduction of preterm delivery emerged as the only significant benefit of supplementation in previous research [2,3].

The present study aims to investigate serum zinc and copper levels in mothers and neonates in relation to prematurity and intrauterine growth retardation, shedding light on potential nutritional interventions to improve outcomes in at-risk neonates.

## Materials and methods

Study design and data collection

This was a cross-sectional study conducted at Dr. D. Y. Patil Medical College, Hospital & Research Centre, Pune, India. The study was approved by the Institutional Ethics Sub-Committee of Dr. D.Y. Patil Medical College, Hospital & Research Centre (approval number: I.E.S.C./W/52 /2024). A total of 100 mothers who met the inclusion criteria (given below) were selected. Before sample collection, patient/parental consent was obtained for the collection of maternal and cord blood samples for zinc and copper analysis. Maternal history was recorded, and gestational age was estimated using the New Ballard scoring system [9]. Birth weights were then plotted against gestational age using Fenton growth charts to determine if the neonates were small or appropriate for gestational age. Physical examinations of the newborns were conducted at birth, and those requiring further medical attention were admitted to the neonatal intensive care unit for appropriate management and monitoring.

Inclusion and exclusion criteria

The study's inclusion criteria encompassed mothers who delivered neonates at or after 32 completed weeks of gestation. Exclusion criteria included infants with congenital malformations or those born from multiple gestation pregnancies, as well as mothers with severe malnutrition (BMI less than 19) or with conditions such as gestational or overt hypertension, gestational or overt diabetes mellitus, chronic illness, severe anemia, or placental abnormalities. These criteria aimed to ensure a specific cohort for the study while excluding potential confounding factors that could affect the outcomes being investigated.

Laboratory testing

Zinc

This study assessed zinc levels using the endpoint nitro PAPS dye-binding method. The principle of this method involves the reaction of nitro PAPS with zinc in an alkaline solution, forming a purple-colored complex. The absorbance of this complex was then measured at 575 nm using a spectrophotometer. The reference value for serum zinc concentration in this study was set between 70-130 microgram per decilitre (μg/dL). The method demonstrated linearity up to 500 μg/dL of zinc concentration. Additionally, the sensitivity of the assay was determined to be 6.92 μg/dL, representing the minimum detectable concentration of zinc with an acceptable level of precision.

Copper

The estimation of copper concentration is conducted by measuring the absorbance of the product formed after reduced Cu^2+^ ions react with a chromogen (3,5-DiBr-PAESA), at 600 nm. The reference value for serum copper was established to be within the range of 95-126 μg/dL. The method exhibits linearity up to 100 micromoles per liter (μmol/L), indicating that the assay can accurately measure copper concentrations within this range. Regarding sensitivity, the minimum detectable concentration of Cu^2+^ with an acceptable level of precision was determined to be 1.97 μmol/L.

Under acidic conditions, ceruloplasmin (CER) bound to Cu^2+^ dissociates, resulting in the formation of free Cu^2+^. Similarly, albumin bound to Cu^2+^ also dissociates under acidic conditions, leading to the release of Cu^2+^ ions. In the presence of a reducing agent, Cu^2+^ ions are reduced to Cuprous ions, which then react with a chromogen (3,5-DiBr-PAESA) to form a blue-colored complex. This complex is then measured to determine the concentration of Cu^2+ ^in the sample.

Statistics

The data entry was done using Microsoft Excel (Microsoft Corporation, Redmond, Washinton, United States), followed by analysis utilizing SPSS for Windows, Version 16.0 (Released 2007; SPSS Inc., Chicago, United States). Qualitative data were represented in frequencies and percentages, while quantitative data were expressed in terms of mean and standard deviation. Nonparametric statistical methods, such as the Chi-square test, were used to determine significant associations between two qualitative variables. Unpaired and ANOVA tests were utilized to assess the statistical significance between quantitative variables. A p-value of less than 0.05 was considered statistically significant.

## Results

The majority of the study population (mothers) was within the age range of 19-28 years, comprising 83% of the total. Specifically, 42 patients (42%) were between 19 and 23 years of age, while 41 patients (41%) were between 24 and 28 years of age. Additionally, there were 15 patients (15%) in the age range of 29-34 years, and only two participants (2%) were older than 35 years. The mean age was 24.76±3.82 years. Among the study population, 49 patients (49%) were primiparous, while 51 patients (51%) were multiparous. This indicates an almost equal distribution between the two parity categories within the sample. Gestational age distribution among the study population was done; 16 women (16%) were in the gestational age range of 32-37 weeks, while the majority, comprising 84 women (84%), were in the range of 38-40 weeks. The mean gestational age for the entire sample was found to be 38.27±1.85 weeks. Among the study population, 16 cases (16%) were classified as preterm pregnancies, while the majority, consisting of 84 cases (84%), were categorized as term pregnancies. This showed a higher prevalence of term pregnancies within the study population. A total of 53 participants (53%) had a normal vaginal delivery (NVD), while the remaining 47 participants (47%) were delivered via lower segment cesarean section (LSCS).

Table 1 illustrates the distribution of birthweights among the neonates delivered by the participants in our study population (n=100) with each delivering a single baby. The mean birthweight for the sample population was calculated to be 2.76 kg, with a standard deviation of 0.51 kg.

**Table 1:** Distribution of neonates based on birthweight (N=100)

Birthweight	Frequency	Percentage
1.5-2 kg	13	13%
2-2.5 kg	14	14%
2.5-3 kg	46	46%
>3 kg	27	27%

Table 2 illustrates the length and head circumference of neonates in the study population.

**Table 2:** Distribution of neonates based on length and head circumference (N=100)

	Minimum	Maximum	Mean SD	95% CI	Median
Length (cm)	46.40	52.50	49.04 ± 1.62	48.17-49.36	48.70
Head Circumference (cm)	33.7	37.80	36.04 ± 0.81	35.88-36.20	36.20

The distribution of birthweights in the studied population revealed appropriate for gestational age (AGA), small for gestational age (SGA), and large for gestational age (LGA). These findings highlight the predominance of infants with birthweights falling within the normal range for their gestational age (Table 3).

**Table 3:** Distribution of neonates according to birthweight associated with age (N=100)

Birthweights	Frequency	Percentage
Appropriate for Gestational Age	70	70%
Small for Gestational Age	27	27%
Large for Gestational Age	3	3%

Table 4 shows the distribution of infants having intrauterine growth restriction (IUGR). This indicates that a majority of infants experienced normal growth during intrauterine development.

**Table 4:** Distribution of neonates based on incidence of IUGR (N=100) IUGR: intrauterine growth restriction

Incidence of IUGR	Frequency	Percentage
Yes	12	12%
No	88	88%

The distribution of maternal zinc levels among the study population is shown in Table 5. The mean maternal zinc level was calculated to be 94.77 μg/dL, with a standard deviation of 12.76 μg/dL (Table 5).

**Table 5:** Distribution of study population based on maternal zinc levels (N=100)

Maternal zinc levels (μg/dL)	Frequency	Percentage
<87.9	20	20%
88 – 130	78	78%
>130	2	2%

The distribution of maternal copper levels among the study population is shown in Table 6. The mean maternal copper level was calculated to be 107.18 μg/dL, with a standard deviation of 11.74 μg/dL.

**Table 6:** Distribution of study population based on maternal copper levels (N=100)

Maternal copper levels (μg/dL)	Frequency	Percentage
<95	15	15%
95 – 126	84	84%
>126	1	1%

The distribution of cord blood zinc levels in the study population is shown in Table 7. The mean cord blood zinc level was calculated to be 138.14 μg/dL, with a standard deviation of 14.93 μg/dL.

**Table 7:** Distribution of study population based on cord blood zinc level (N=100)

Cord blood zinc levels	Frequency	Percentage
<124	12	12%
124 – 160	87	87%
>160	1	1%

The distribution of cord blood copper levels in the study population is shown in Table 8. The mean cord blood copper level was calculated to be 57.82 μg/dL, with a standard deviation of 18.65 μg/dL.

**Table 8:** Distribution of study population based on cord blood copper level (N=100)

Cord blood copper levels (μg/dL)	Frequency	Percentage
<40	24	24%
40 – 90	73	73%
>90	3	3%

The comparison of maternal zinc levels between term and preterm neonates revealed a statistically significant difference. The difference was found to be statistically significant (Table 9).

**Table 9:** Association of maternal zinc and copper level and gestational age *p-value less than 0.05 is considered significant

	Term	Preterm	p-value
Maternal zinc levels (μg/dL)	95.95 ±12.80	88.55 ± 10.90	p = 0.03*
Maternal copper levels (μg/dL)	111.03 ± 8.12	86.97 ± 5.16	p<0.0001*

The comparison of maternal zinc and copper levels among neonates classified as AGA, SGA, and LGA revealed a statistically significant difference (Table 10).

**Table 10:** Association of maternal zinc and copper level and birthweight according to age AGA: appropriate for gestational age; SGA: small for gestational age; LGA: large for gestational age * p-value less than 0.05 is considered significant

	AGA	SGA	LGA	p-value
Maternal zinc (μg/dL)	95.49 ±12.50	92.31 ± 13.89	100.16 ±4.00	0.02*
Maternal copper levels (μg/dL)	110.87 ± 7.88	97.06 ± 14.05	112.23 ±14.58	0.0001*

The comparison of maternal zinc and copper levels between mothers with neonates diagnosed with IUGR and those without IUGR showed a statistically significant difference (Table 11).

**Table 11:** Association of maternal zinc and copper levels and incidences of IUGR * p-value less than 0.05 is considered significant IUGR: intrauterine growth retardation

	Yes	No	p-value
Maternal zinc levels (μg/dL)	84.78 ±12.39	95.58 ± 12.66	0.006*
Maternal copper levels (μg/dL)	85.63 ± 4.96	110.12 ± 9.20	< 0.0001*

The comparison of mean cord blood zinc and copper levels between neonates with IUGR and those born without IUGR revealed a statistically significant difference (Table 12).

**Table 12:** Association of cord zinc and copper levels and incidences of IUGR * p-value less than 0.05 is considered significant IUGR: intrauterine growth restriction

	Yes	No	p-value
Cord blood copper levels (μg/dL)	115.23 ±15.48	141.26 ± 11.88	0.001*
Cord blood copper levels (μg/dL)	33.71 ± 4.10	61.11 ± 17.40	0.001*

## Discussion

The trace elements, such as zinc and copper are critical in human growth and development [10]. They play crucial roles in various physiological processes, serving as essential components for transcription factors and catalytic cofactors for enzymes involved in cell differentiation and maturation [11]. Zinc, for example, acts protectively against free radicals and is integral to intracellular processes like cell division and nucleic acid synthesis. Similarly, copper is a vital cofactor for oxidative and reductase enzymes [12].

Despite their significance, deficiencies in these trace minerals are not uncommon, particularly among preterm infants who may not receive adequate intrauterine fetal growth-related nutrients. Thus, understanding maternal mineral status during gestation becomes imperative for ensuring fetal and neonatal health.

In this study, we found that maternal copper and zinc levels were found to be significantly less in the cases of preterm birth when compared to term birth cases. A study by Nawfal et al. indicates a significant association between lower serum copper levels in mothers and a higher risk of developing PPROM [13]. Another study by Monangi et al. shows a negative association between maternal copper levels and gestational duration, as well as a positive association with the risk of preterm birth [14]. This suggests that higher maternal copper levels are linked to shorter gestational periods and an increased likelihood of experiencing preterm birth. In contrast, another study by Hao et al. suggests that elevated maternal copper levels during the first trimester of pregnancy could potentially heighten the risk of spontaneous preterm birth [15]. Several other studies have consistently shown the association of maternal serum copper and zinc levels with gestational age [16].

In this study, we found that maternal and cord blood cooper and zinc levels were significantly lower in the IUGR group. A similar study by Çelik et al. revealed significant differences in placenta zinc concentrations and the placenta zinc/copper ratio between the IUGR group and the control group, with the IUGR group exhibiting lower levels (p < 0.05) [17]. Furthermore, there was a notable correlation observed between placenta zinc concentrations and birth weight (p: 0.01, r: 0.31), suggesting a potential role of zinc in fetal growth. These results underscore the importance of adequate placental zinc levels in supporting optimal fetal development, with deficiencies possibly contributing to the development of IUGR. Abass et al., in their comparison study of maternal zinc and copper levels along with cord copper levels, reveal notable differences between newborns LBW and those with normal weight. Specifically, LBW newborns exhibit lower zinc and copper levels than their normal-weight counterparts. Additionally, cord copper levels are also diminished in LBW infants. These findings suggest a potential association between maternal trace element status and fetal growth [18]. Several other studies have attributed maternal zinc and copper levels to IUGR [19-21].

Although there have been studies on maternal and cord blood levels of zinc and copper, findings have been inconsistent. Therefore, this study aimed to measure zinc and copper levels in both maternal and cord blood samples and correlate these levels with gestational age and anthropometric measurements. Such investigations are crucial for providing healthcare professionals with essential data for appropriate management strategies.

Limitations

One significant limitation of this study is its relatively small sample size, which may impact the generalizability and reliability of the findings. Increasing the population size would enhance the statistical power of the study and allow for a more robust interpretation of the results. Additionally, this study only considered post-delivery cases, which means that it overlooks various intrauterine parameters that could potentially influence the outcomes. Including data on intrauterine parameters would provide a more comprehensive understanding of the factors contributing to the observed results.

## Conclusions

This study highlights the critical role of trace elements, particularly zinc and copper, in fetal development and neonatal health. Our findings reveal significantly lower levels of these elements in maternal and cord blood in cases of preterm birth and IUGR. These deficiencies are linked to adverse outcomes, emphasizing the need for adequate maternal nutrition during pregnancy to support optimal fetal growth. The association between lower trace element levels and conditions like preterm birth and IUGR suggests that current nutritional guidelines and supplementation strategies may require reassessment to better address the needs of expectant mothers and their babies.

Understanding the exact mechanisms by which these trace elements influence fetal development and identifying the optimal levels required for healthy growth can help healthcare professionals develop more effective management strategies. Continued research efforts are crucial to refine supplementation guidelines and improve outcomes for preterm neonates and those at risk of IUGR.

## References

[1] Jana A, Saha UR, Reshmi RS, Muhammad T (2023). Relationship between low birth weight and infant mortality: evidence from National Family Health Survey 2019-21, India. Arch Public Health.

[2] Barros FC, Rossello JL, Matijasevich A (2012). Gestational age at birth and morbidity, mortality, and growth in the first 4 years of life: findings from three birth cohorts in Southern Brazil. BMC Pediatr.

[3] Ilardi L, Proto A, Ceroni F, Morniroli D, Martinelli S, Mosca F, Giannì ML (2021). Overview of important micronutrients supplementation in preterm infants after discharge: a call for consensus. Life (Basel).

[4] MacDonald RS (2000). The role of zinc in growth and cell proliferation. J Nutr.

[5] Black MM (1998). Zinc deficiency and child development. Am J Clin Nutr.

[6] Guan L, Wang Y, Lin L, Zou Y, Qiu L (2024). Variations in blood copper and possible mechanisms during pregnancy. Biol Trace Elem Res.

[7] Gromadzka G, Tarnacka B, Flaga A, Adamczyk A (2020). Copper dyshomeostasis in neurodegenerative diseases-therapeutic implications. Int J Mol Sci.

[8] Tomat A, Elesgaray R, Zago V (2010). Exposure to zinc deficiency in fetal and postnatal life determines nitric oxide system activity and arterial blood pressure levels in adult rats. Br J Nutr.

[9] Ballard JL, Khoury JC, Wedig K, Wang L, Eilers-Walsman BL, Lipp R (1991). New Ballard Score, expanded to include extremely premature infants. J Pediatr.

[10] Wróblewski M, Wróblewska J, Nuszkiewicz J, Pawłowska M, Wesołowski R, Woźniak A (2023). The role of selected trace elements in oxidoreductive homeostasis in patients with thyroid diseases. Int J Mol Sci.

[11] Tapiero H, Townsend DM, Tew KD (2003). Trace elements in human physiology and pathology. Copper. Biomed Pharmacother.

[12] Jomova K, Makova M, Alomar SY (2022). Essential metals in health and disease. Chem Biol Interact.

[13] Nawfal H, Alhamid A, Abd Al-Jawad AA (2020). Correlation between maternal copper deficiency and premature rupture of membranes: a case-control study. Libyan Int Med Univ J.

[14] Monangi NK, Xu H, Fan YM (2024). Association of maternal prenatal copper concentration with gestational duration and preterm birth: a multicountry meta-analysis. Am J Clin Nutr.

[15] Hao Y, Pang Y, Yan H (2019). Association of maternal serum copper during early pregnancy with the risk of spontaneous preterm birth: a nested case-control study in China. Environ Int.

[16] Gohari H, Khajavian N, Mahmoudian A, Bilandi RR (2023). Copper and zinc deficiency to the risk of preterm labor in pregnant women: a case-control study. BMC Pregnancy Childbirth.

[17] Yücel Çelik Ö, Akdas S, Yucel A, Kesikli B, Yazihan N, Uygur D (2022). Maternal and placental zinc and copper status in intra-uterine growth restriction. Fetal Pediatr Pathol.

[18] Abass RM, Hamdan HZ, Elhassan EM, Hamdan SZ, Ali NI, Adam I (2014). Zinc and copper levels in low birth weight deliveries in Medani Hospital, Sudan. BMC Res Notes.

[19] Begum S, Mahmood S (2024). Association of maternal serum zinc status with neonatal birth weight. Int J Reprod Contracept Obstet Gynecol.

[20] Mukherjee MD, Sandstead HH, Ratnaparkhi MV, Johnson LK, Milne DB, Stelling HP (1984). Maternal zinc, iron, folic acid, and protein nutriture and outcome of human pregnancy. Am J Clin Nutr.

[21] Tamura T, Goldenberg RL, Freeberg LE, Cliver SP, Cutter GR, Hoffman HJ (1992). Maternal serum folate and zinc concentrations and their relationships to pregnancy outcome. Am J Clin Nutr.

